# The silent transition from curative to palliative treatment: a qualitative study about cancer patients’ perceptions of end-of-life discussions with oncologists

**DOI:** 10.1007/s00520-020-05750-0

**Published:** 2020-09-12

**Authors:** A. Kitta, A. Hagin, M. Unseld, F. Adamidis, T. Diendorfer, E. K. Masel, K. Kirchheiner

**Affiliations:** 1 Clinical Division of Palliative Care, Department of Internal Medicine I, Medical University of Vienna, Waehringer Guertel 18-20, 1090 Vienna, Austria; 2 Faculty of Psychology, University of Vienna, Liebiggasse 5, 1010 Vienna, Austria; 3 Department of Radiation Oncology, Medical University of Vienna, Waehringer Guertel 18-20, 1090 Vienna, Austria

**Keywords:** Communication, Physician-patient relationship, Advanced care planning, End-of-life discussions, Terminal care, Qualitative research

## Abstract

**Objective:**

The aims of the study were to examine patients’ experiences of end-of-life (EOL) discussions and to shed light on patients’ perceptions of the transition from curative to palliative care.

**Methods:**

This study was based on a qualitative methodology; we conducted semi-structured interviews with advanced cancer patients admitted to the palliative care unit (PCU) of the Medical University of Vienna. Interviews were recorded digitally and transcribed verbatim. Data were analyzed based on thematic analysis, using the MAXQDA software.

**Results:**

Twelve interviews were conducted with patients living with terminal cancer who were no longer under curative treatment. The findings revealed three themes: (1) that the medical EOL conversation contributed to the transition process from curative to palliative care, (2) that patients’ information preferences were ambivalent and modulated by defense mechanisms, and (3) that the realization and integration of medical EOL conversations into the individual’s personal frame of reference is a process that needs effort and information from different sources coming together.

**Conclusions:**

The results of the present study offer insight into how patients experienced their transition from curative to palliative care and into how EOL discussions are only one element within the disease trajectory. Many patients struggle with their situations. Therefore, more emphasis should be put on repeated offers to have EOL conversations and on early integration of aspects of palliative care into the overall treatment.

**Electronic supplementary material:**

The online version of this article (10.1007/s00520-020-05750-0) contains supplementary material, which is available to authorized users.

## Introduction

The end of curative treatment is not the end of treatment options in the medical system. Palliative care aims to relieve symptoms and improve quality of life without curing the underlying disease [1]. The transition from curative to palliative treatment is a challenging process that evolves over time. However, starting palliative care earlier in the progression of the disease has been proven to benefit patients [2, 3]. In oncological palliative care, there might be a clear cut-off for medical professionals to end curative treatment, such as the spread of disease, the deterioration of organ function, or the performance status of the patient. However, these cut-off points might not have the same meaning to the person undergoing treatment. For example, stage IV cancer might be treated with chemotherapy, which is clearly on a palliative scale for the oncologist but not necessarily for the patient [4]. It is therefore mandatory for medical professionals to guide patients through this transition. A part of this transitional process are end-of-life (EOL) discussions, which offer opportunities for shared decision-making and discussing future goals [5]. However, with advances in modern oncology, therapeutic options have become numerous and have blurred the line between curative and palliative treatment. This can lead to EOL discussions being delayed [6, 7].

Through shared decision-making, physicians and patients are meant to become partners in the process [8]. The benefits thereof have been studied extensively [9–12] and include higher patient satisfaction, better therapeutic outcomes, and increased quality of life [8, 13]. In addition, patients’ preferences regarding the quantity and quality of information within an EOL conversation have been evaluated in previous studies [14–16]. However, few reports have focused on patients’ retrospective reflections on EOL discussions in order to be able to investigate patients’ experiences. Thus far, no studies done in Austria have asked patients to reflect on EOL discussions or assessed patients’ experiences [17–19]. The aim of this study was therefore to examine how patients experienced the transition from curative to palliative care and how patients perceived EOL discussions.

## Materials and methods

### Interview guide

Due to the explorative nature of this topic, a qualitative study design with semi-structured interviews was used. The qualitative method allowed participants to elaborate on whatever they thought was important while providing a frame of orientation [20]. Therefore, predetermined open-ended questions were collected in an interview guide (Appendix Table 1). The interviewer started every interview by asking participants for their medical history and closed the interview by asking about expectations regarding their future. If the participants were in a state of denial, no further confrontation was forced.

### Participants and data collection

﻿The study was performed in the palliative care unit (PCU) of the Medical University of Vienna, which is a hospital-based facility consisting of 12 beds mainly reserved for cancer patients, who are admitted or transferred through suggestion of oncologists. Approximately 40% of the patients are being discharged from the PCU after receiving best supportive care.

Inclusion criteria for the study were age over 18 years; no severe reasons for non-participation such as cognitive ﻿deficit, delirium, mental illness, or septicemia; no language problems; has the ability to give written informed consent; and that the patient was hospitalized at the PCU for at least 36 h so that the personnel could judge the physical and mental state of the patient. ﻿Patients had to have terminal cancer that was no longer under curative treatment and were assessed by their oncologist with the prognosis of a 2- to 12-month life expectancy and had an EOL discussion prior to admission. Patients who suffered from a psychiatric diagnosis such as dementia or had brain tumors or brain metastasis were excluded from the study, as recollection abilities were vital to the interview process. ﻿To minimize bias, all patients were interviewed by one female psychology student (AH) who was not part of the PCU staff. The qualitative study was approved by the institutional ethics committee (1121/2018).

### Data analysis

The study was conducted following the COREQ-32 criteria for qualitative studies [21]. The interviews were recorded digitally and transcribed verbatim [22]. They were conducted in German, and citations in the present report were translated by a professional translator. Thematic analysis was applied as it allows analyzing descriptions of experiences [23–25]. ﻿﻿The coding process started after the transcription of the first interviews and was continued throughout the entire analysis process. Data were analyzed with the software MAXQDA. Three researchers (AK, AH, KK) independently generated a list of codes inductively out of the participants’ answers after reading the first interviews. The results were then compared. There was a high level of agreement between the encoders, and minor differences were resolved by group discussion with two other researchers (EKM, TD). When theoretical saturation was reached (i.e., the analysis of the most recent interview did not generate new codes), one further interview was carried out for confirmation. As no further codes were identified, the interview process was finished. Themes were identified, reviewed, defined, and named by consensus in the team (Appendix Table 4).

## Results

### Patients’ characteristics

**Fig. 1:**
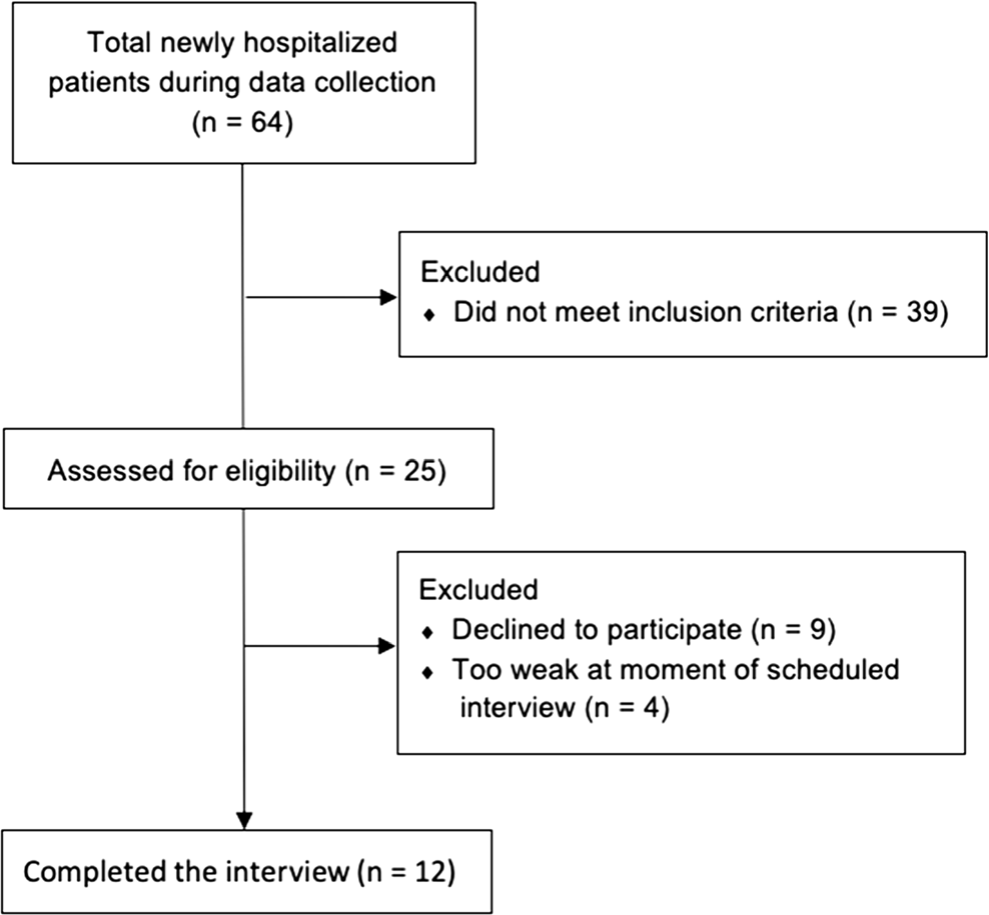
Consort diagram of included patients

During the 5 months of data collection, 64 patients were hospitalized in the PCU, and 25 of them fulfilled the inclusion criteria for our study. These patients were consecutively invited to participate, and interviews were then conducted with twelve (7 female and 5 male, Fig. 1). The duration of the interview ranged from 14 to 41 min (mean 28 min), and patients were between 49 and 91 years of age (mean 69 years). Two patients underwent treatment, entered remission, and experienced a relapse. Taking that relapse as a new point of reference, patients had been in treatment prior to the interview for an average of 26 months (range 3–44 months). Sociodemographic characteristics are shown in Appendices Tables 2 and 3. The original German quotations are available in Supplementary Information (S1 Appendix).

### Qualitative findings

#### Theme 1: Contribution of the medical EOL conversation to the transition process from curative to palliative care

Some patients identified a single conversation with their oncologist who treated them as having changed their personal perspective. These EOL conversations were described as short (P06, P10), and patients felt the time pressure of the physicians (P04). Patients experienced difficulties asking questions for various reasons, such as that the oncologists continued speaking right after breaking the bad news and patients were too nervous or needed time to realize what had been said (P01, P05, P10). It was reported that the physicians switched immediately from the bad news to more manageable topics: “she [the oncologist] said ‘There is nothing left. We have tried everything’” and “that she is sorry, but [...] now one could only wait and see. […] Then she immediately went on with the bone scan [...] and then referred me to the radiation clinic” (P05). Patient 6 had contacted another oncologist after the conversation with her oncologist for a second opinion. She remembered: “They both said only: ‘I am sorry, I can’t do anything else for you.’ Both the same words. Really, I was surprised. […] In this moment, neither of the physicians spoke much.” For patient 10, this kind of briefness was rather hurtful: “But after he had said in which situation I am, the matter was finished relatively quickly for Prof. [oncologist]. It was like, ‘Now I told you, that is how it is. Goodbye.´”

However, other interviewees experienced an avoidance of clear communication. Patient 7 was told that a “break” was planned, and the oncologist explained, “There was no bed available in the oncological unit. And he [the oncologist] said, that at the palliative care unit it would be very nice, and I’d be fine.” Another patient reported some disappointment with a lack of details in the EOL discussion: “I wanted to know everything. I wanted to know in detail, how long I have to live or how long it can go on, because she [the oncologist] can´t tell me precisely […] They don´t like to specify, the doctors, right?” (P05).

The interviews revealed that the majority of patients had negative attitudes toward PCUs, with at least some patients linking PCUs explicitly to dying prior to their admission to the ward. ﻿Some oncologists seemed to be aware of the preconceptions toward the word “palliative” and had therefore decided to use euphemisms and mention how positively other patients had responded to palliative care. Patient 10 repeated the words of his oncologist: “﻿’We have a palliative care unit where you are going to be pampered. Patients enjoy it a lot, the team there is very nice, competent.’” However, according to the majority of the participants, ﻿physicians did not explain either the concept or the purpose of a palliative care unit, and thus patients drew their own conclusions (e.g., P03, P07).

In summary, the EOL information provided by oncologists was experienced as brief and concise, with a tendency for physicians to switch to other topics without addressing the subjective experience of their patients. Therefore, patients may not have been able to come to terms with the reality of their illness being terminal.

#### Theme 2: Patients’ information preferences were ambivalent and modulated by defense mechanism

Patients’ expectations for the future and the immediate next steps were open and vague, as shown by patient 1. He stated that he wanted no further chemotherapy and explained, “Now we are waiting for results. And then we will see how we will continue with therapy.” Patient 7’s answer to the interviewer’s question about whether she would get another round of chemotherapy was “No idea.” Also, patients 10 and 11 mentioned that they were currently waiting for a further procedure. Patient 12 was not even sure how long she was able to stay at the PCU: “I do not know how long they are going to keep me.” Similarly, patient 4 mentioned, “Nobody really explained the next steps to me.”

In retrospect, we cannot know whether there was a lack of information or if patients distanced themselves from the realization of their illness’s incurability as a sign of defense mechanisms. However, ambivalence about wanting information on the one hand and refusing information on the other hand was prevalent in some patients, including patient 9. Throughout the interview, she stated a few times that she had not wanted information from her oncologist with regard to her disease: “I never asked. I did not want to know it” and “Nobody told me anything.” In regard to her prognosis, she later admitted that a physician from the radiation department had told her about the severity of her situation, even if she did not want to know about that. Ambivalence was also shown in statements of other participants who initially said that they wanted to know everything (e.g., P03, P05, P07) but who also gave contrary statements in the very same interview.

This might reflect a defense mechanism described in the literature as “middle knowledge,” a fluctuation between active realization and resistance. Patient 2, for example, expressed a clear realization about the fact that his illness was incurable but immediately questioned it again: “I know, how bad off I am [and] what time has come. When I heard ‘palliative care unit’, I thought, ‘Oh boy. End of line.’ But that is not how it is.”

Patient 10 perceived himself as not being in the right place: “[I am not] a real palliative care patient, because I am still in therapy and not, as you would say, beyond treatment.” Similarly, patient 11, who had only been diagnosed 3 months earlier, hardly used the word “cancer” throughout the interview and concluded with “﻿I do not want to be here. I am not dealing with that at the moment,” showing tendencies toward suppression and denial.

On the other hand, acceptance of imminent death was also expressed. Three patients explained their acceptance with a life review during the interview (P03, P08, P12). ﻿As patients 3 and 12 were 79 and 91 years of age, respectively, the presentation of a fulfilled life seemed comprehensible, but 55-year-old patient 8 revealed acceptance going beyond the years of life: “Look, when you have had a past like me, nothing surprises you anymore. Sixties, sex, drugs and rock n’ roll. For real. […] I know that I can die at any time. The advantage for me is that I don't know when I will die. […] That is the nice thing. No time limit. So, from there ... Everything easy.”

In conclusion, because patients showed defense mechanisms toward EOL, the disease trajectory from curative to palliative care was not always clearly described by patients. With various levels of realization about the severity of their disease, information preferences were often ambivalent.

#### Theme 3: Realization and integration of medical EOL conversations into the individual’s personal frame of reference is a process that needs effort and information from different sources coming together

﻿In order to process and integrate EOL realization into a personal and meaningful frame of reference, patients used additional sources of information. Some patients took advantage of the Internet to look up information regarding their disease and prognosis (P01, P03, P10). Others reported conversations with friends or family members with medical knowledge as a resource. Patient 5 benefited from his wife, who worked for a pulmonologist*:* “She can read these whole results, […] and she knew what was coming.” Others mentioned personal experiences with people suffering from cancer, such as patient 3: “Of course, I looked up what it all meant on the internet. But I knew it beforehand, as already three of my friends died of this cancer.” Some drew conclusions from conversations with other patients whom they met in waiting areas (P01, P09). And patient 7 ﻿stated that she picked up a lot from the media: “Every time you hear about cancer [be it] on television, in the newspaper, of course, you pay special attention and read that very carefully.” On the contrary, some patients deliberately did not search for additional information and saw no need for it (P08, P10).

In addition, the participants drew conclusions from observations of their own physical condition. For patient 6, being attentive to her body started very early in her disease trajectory: “I felt a lump here. And I already knew I had cancer.” In contrast, due to the loss of her ability to go for a run on her favorite trail, patient 4 became aware of her restricted lifetime on the day of her arrival on the PCU: “suddenly it came to my mind ‘You will never go back there’… And that physical limitation [suppresses crying] shook me badly on the first day when I was here.”

The moment of becoming fully aware of their palliative status and their own mortality emerged like individual puzzle pieces coming together, revealing the view of a bigger picture. This realization was described by patients as a stepwise process, and the medical EOL conversation, in many cases, represented only one puzzle piece. Patient 5 highlighted that once the terminal prognosis has been disclosed, the understanding took some time: “No, you don´t hear that. That … takes two or three days… It comes back to your mind and then you think ‘Oh, I could have asked that.’ […] But yourself, you are overwhelmed with the disease.”

In summary, the interviews revealed that the patients sought out a great deal of information by themselves and also drew their own conclusions based on their physical condition. Realizing and integrating EOL conversations is a process that takes time and effort for the individual. Furthermore, it needs different information sources coming together.

## Discussion

﻿The qualitative results of our study demonstrate the need for greater focus on EOL conversations and more compassionate direction from oncologists. The patients perceived EOL discussions as mostly focusing on medical issues such as types of tests or treatments and therefore searched for additional information by themselves (e.g., what could be expected in the future). The interviews revealed that the disease trajectory and the conversation about prognosis and survival are challenging for both patients and physicians. A relationship of trust is certainly beneficial in this context [26]. This is particularly true given that defense mechanisms may be employed by patients as a vital tool, enabling them to keep functioning after a tremendous threat such as a terminal prognosis. Once the immediate shock has subsided, a person may replace it with more functional coping strategies such as positive reframing and acceptance [27]. Due to the inclusion criteria (having knowledge about the incurability of their cancer and being assessed as physically and mentally stable enough for an interview), the participants of this study represented a group of patients whose transition was—at least to some extent—successful. In this light, the finding that in many cases the EOL discussion was reportedly mostly focused on medical issues might be due to repression to some extent. However, this is also a strong indication that such conversations need training and should be improved [28, 29]. Interview participants mainly named the oncologist as the preferred source of information, which emphasizes the need for more time to be allocated for EOL discussions.

### Difficulties regarding EOL conversations

Even if previous studies described very high numbers of patients desiring information, there are still patients who do not wish to be informed [30, 31]. Within the interviews, it was obvious that some patients did not want their physicians to speak to them about their current situation and prognosis. None of the participants reported having been asked how much information they desired. Taking this into account, it must be emphasized that a single conversation is most likely insufficient to explain all the essential aspects related to the incurability of a disease. Physicians should ideally offer information repeatedly in order to make sure that patients can adapt to their new realities. Physicians might be reluctant to hold EOL discussions, because of prognostication difficulties, reluctance or conflicts within the patients’ family, concerns about patient disappointment, or even feelings of guilt [32]. Mack and Smith [33] outlined different reasons for physicians’ reluctance around sharing the news of a poor prognosis and refuted each reason, providing scientific evidence disproving the concerns raised. However, research on delivering bad news has shown that physicians are less reluctant to discuss various options for care and treatments they themselves can provide than they are to discuss palliative care issues [34].

Therefore, the results of the current study underline the necessity of cooperating in a multidisciplinary team (e.g., oncology and palliative care teams) while taking into account the current preferences, situation, and knowledge of the patients.

### Benefits of EOL conversations

It has been shown that shared decision-making in the context of EOL discussions increases patients’ satisfaction, leading to better therapeutic outcomes and an increased quality of life [8, 13]. Early EOL communication with cancer patients (before their last 30 days of life) results in less aggressive EOL care (such as chemotherapy or admittance to the emergency department) and to a significant increase in receiving hospice care [6, 35]. A common fear of physicians who avoid EOL conversations is the fear of destroying hope [33]. In this regard, Enzinger et al. showed that prognostic conversations led to substantial benefits without harming patients’ emotional well-being or the patient-physician relationship [36]. Fallowfield et al. provide evidence in their study that over 80% of the participants wanted “as much information as possible, good and bad” [30]. Despite this desire for information, patients rarely requested it, as they expected their doctors to provide this kind of information [37]. Rumpold et al. investigated the information preferences of cure rates and prognosis in advanced lung cancer patients in Austria and found that 76% of patients desired information about either cure rates alone or cure rates and prognosis [31]. Therefore, the integration of EOL conversations into daily practice should be fostered continuously [38].

The fact that patients need more information was also observed in the present study. Participants reported a lot of misconceptions, doubt, and fear in regard to being admitted to the PCU. Early information about the PCU might also facilitate an easier transition to the PCU for patients.

## Strengths and limitations of the study

﻿﻿Conducting interviews on the topic of ending curative treatment within a palliative care setting is challenging and requires a high degree of sensitivity to avoid patient distress [39]. The present study confirmed patients’ willingness to participate in a qualitative study [40]. A strength of this study is its response rate of 48% (see Fig. 1) given the study’s topic and general tendency to avoid the subject of death and dying. A limitation of the study might be that patients were recruited from a single, tertiary center. Therefore, the results may not be generalizable to different settings.

## Clinical recommendations

Physicians should provide repeated initiatives for EOL discussions. Furthermore, it is recommended that physicians regularly ask for the individual’s preference for information in regard to EOL topics. Doing so will make it possible to adapt the conversation in the face of momentary coping or defense mechanisms. More emphasis should also be given to the integration of EOL information into the patients’ shifting internal reality after EOL discussions; this might be accomplished by reassuring the patients and asking them about their feelings.

## Conclusions

In EOL conversations, patients described their physicians as being hesitant and evasive and described the conversations themselves as being short, but there was also a certain ambivalence in their own preferences for whether or not to receive information.

Becoming aware of one’s own palliative situation is a step-by-step process. This transition is not possible through a one-point and one-time EOL conversation. In addition to the information received through the EOL discussion, patients looked for information from a conglomerate of other sources. They put this information together like a puzzle over time and were often left to do so alone. Therefore, scheduling of conversations might be important for patients to be able to address all their questions and to have time to come to terms with their own situation.

### Electronic supplementary material

ESM 1 (PDF 450 kb)

## Data Availability

Transcription of the anonymized interviews can be made available upon request by the corresponding author.

## Appendix

**Table 1:** Interview guide

Structural questions (asked every time)	Optional questions (asked depending on what the individual answers had already covered, taking into account the health situation and endurance of the patient)
Can you remember why you went to the physician at the very beginning? Can you remember the moment you heard for the first time that you have cancer?	
What did the doctor tell you regarding the curability of your cancer?	
Was there a point when you realized that your cancer might not be curable? How was this moment for you?	
	Was there anything about this conversation/process that you found particularly stressful?
	Was there anything in this conversation/process that you found particularly helpful?
	Did you know what to expect before the conversation? Where did these expectations come from?
Did you get information about how your disease might progress independently?	
What did you do immediately after the conversation?	
	What did/would you have needed at that moment?
Did you get a follow-up medical appointment? With whom (oncologist, palliative care doctor)? What can you tell me about it?	
Did you have any questions later? Were you able to ask them?	
	Have you been offered, or have you used psychological support or spiritual support?
Was there a moment when you realized that your disease is incurable? How was this moment for you?	
Did you want this kind of information? How did you obtain information?	
When did you come to the palliative care unit? How was that? What does palliative care mean to you? When did you hear the word for the first time?	

**Table 2:** Profile of the study participants

Participants	Age (years)	Sex	Type of cancer	Previous therapies	Duration since first diagnosis (duration since relapse)	Duration of interview (min)
P01	67	M	Pancreatic	Surgery, chemotherapy	3 years and 8 months	29
P02	79	F	Sarcoma	Radiotherapy, chemotherapy	2 years and 4 months	34
P03	79	M	Lung	Radiotherapy, chemotherapy, immunotherapy	3 years and 2 months	14
P04	55	F	Pancreatic	Chemotherapy	6 months	20
P05	55	M	Lung	Radiotherapy, chemotherapy, immunotherapy	2 years and 4 months	18
P06	88	F	Breast	Radiotherapy, chemotherapy, surgery	18 years and 5 months (3 years and 8 months)	31
P07	79	F	Breast	Radiotherapy, chemotherapy, surgery	29 years and 3 months (2 years and 6 months)	29
P08	55	M	Hypopharynx	Radiotherapy, chemotherapy, immunotherapy	3 years and 8 months	17
P09	64	F	Colorectal	Surgery	1 year and 9 months	41
P10	57	M	Urothelial	Chemotherapy, surgery, immunotherapy	1 year and 5 months	35
P11	49	F	Lung	Chemotherapy	3 months	33
P12	91	F	Ovarian	None	7 months	32

**Table 3:** Baseline characteristics of patients

Interview participants, *n* = 12			
		*n*	%
Sex	Female	7	58
	Male	5	42
Marital status	Married	7	58
	Widowed	3	25
	Divorced	1	8
	Single	1	1
Children	Yes	10	83
	No	2	17
Histology	Lung cancer	3	25
	Pancreatic cancer	2	17
	Breast cancer	2	17
	Sarcoma	1	8
	Hypopharyngeal cancer	1	8
	Colorectal cancer	1	8
	Urological cancer	1	8
	Ovarian cancer	1	8
Treatments received	Chemotherapy	10	83
	Radiotherapy	6	50
	Surgery	5	42
	Immunotherapy	4	33
Age	Mean	Median	Range
	69	65.5	49–91

**Table 4:** Themes, categories, and codes generated from the patient interviews

Topic	Themes	Categories	Examples for codes
Patient’s perception of their transition from curative to solely palliative treatment	Contribution of the medical EOL conversation to the transition process from curative to palliative care	EOL discussion	Setting Physician’s behavior Definition of PCU by physician Recalling of exact wordings Physician’s avoidance Pos. aspects of communication Neg. aspects of communication Opportunity to ask questions
		Realization through admittance to PCU	Perception of PCU Conversations about PCU
	Patients’ information preferences were ambivalent and modulated by defense mechanism	Information preferences	Aversion Passive reception Active procurement
		Perception of future	Further therapies Remaining lifetime Unspeakable aspects Not knowing
		Underlying coping mechanisms	Defense mechanisms Denial Middle knowledge Positive thinking and reframing Acceptance
	Realization and integration of medical EOL conversations into the individual’s personal frame of reference is a process that needs effort and information from different sources coming together	Active information procurement	Internet TV/films Books Acquaintances/family/friends Shared decision-making
		Self-experience	Self-assessment Own will and decisions Body knowledge Experiences of earlier observation of cancer diseases in surroundings
