# Supplementary material for: The silent transition from curative to palliative treatment: a qualitative study about cancer patients’ perceptions of end-of-life discussions with oncologists
Source: Support Care Cancer. 2020 Sep 12;29(5):2405–13. doi: 10.1007/s00520-020-05750-0 (PMC7981304; doi:10.1007/s00520-020-05750-0)
Supplement: Supplementary file 1 — (PDF 450 kb) [file 520_2020_5750_MOESM1_ESM.pdf]

Appendix
S1. Original German quotations in order of appearance in the text.

Theme 1

P05: „Da hat sie dann gesagt: ‚Es gibt nichts mehr. Wir haben alles ausprobiert.'"

„Ja, dass es ihr halt leid tut, aber [...] jetzt kann man halt nur mehr schauen... Ja... das es halt so lang wie möglich geht, nicht? Dann hat sie sofort [...] gesagt, da machen wir gleich eben das Knochenscan da [...] und dann hat sie mich verwiesen auf die Strahlenambulanz"

P06: „Die haben beide nur gesagt: ‚Es tut mir leid, ich kann nichts mehr für Sie tun.' Jeder dieselben Worte. Wirklich, das hat mich gewundert. [...] Da haben beide Ärzte, egal welcher es war, nicht viel gesprochen."

P10: „Aber nachdem das heraussen war, in welcher Situation ich mich befinde, war für den Herrn Primar [Onkologe] die Sache relativ schnell zu Ende gesprochen. Das heißt: ‚Ich habe das jetzt gesagt, so ist es, auf Wiedersehen.'"

P07: „Der Arzt sagt nur, dann ... machen wir Pause."
„Na ich glaube auf der Onkologie, glaube ich war nichts frei. Und er hat gemeint da auf der Palliativ, da ist es sehr schön und da wird es mir sehr gut gehen."

P05: „Ich wollte alles wissen. Ich wollte genau wissen, wie lange lebe ich noch oder wie lang kann es noch gehen, weil genau kann sie es mir ja nicht sagen. Aber... da legen sie sich halt nicht gerne fest, die Ärzte, nicht?"

P10: „Wir haben eine Palliativstation, so dass, die Sie aufpäppeln. Das kommt unheimlich gut an, die sind sehr nett, kompetent und und und."

Theme 2

P01: „Jetzt warten wir auf die Befunde. Und dann schauen wir wie es weiter geht mit der Therapie."

P07: „Keine Ahnung."

P12: „Ich weiß nicht, wie lange sie mich behalten."

P04: „Es hat mir eigentlich niemand großartig die weiteren Schritte erklärt."

P09: „Ich habe nie gefragt. Ich wollte das nicht wissen."
„Das hat mir nie jemand gesagt."

P02: „Ich weiß, dass ich schlecht dran bin [und] ich weiß eh was es geschlagen hat. Wie ich gehört habe, Palliativstation habe ich mir gedacht: "Na Servus. Endstation." Aber es ist ja nicht so."

P10: „Nicht als richtiger Palliativpatient, weil ich ja noch in Behandlung bin und nicht, dass du sagst, austherapiert."

P11: „Ich will da gar nicht her. Mit dem setze ich mich jetzt momentan überhaupt nicht auseinander."

P08: „Schauen Sie, wenn du eine Vergangenheit gehabt hast wie ich, dann überrascht dich nichts mehr. 60er Jahre, Sex, Drugs und Rock 'n Roll. Tatsächlich. [...] Ich weiß, dass ich

jederzeit sterben kann. Der Vorteil bei mir ist, ich weiß nicht wann ich sterbe [...] Das ist das Schöne. Keine Befristung. Also von daher... Alles easy.“

Theme 3

P01: „Sie kann diese ganzen Befunde lesen [...] und sie hat schon gewusst was auf sie zukommt.“

P03: „Ich hab natürlich im Internet nachgelesen was das Ganze bedeutet. Wusste es aber vorher schon, weil es sind schon, drei meiner Bekannten eben diesem Krebs gestorben.“

P07: „Eben, immer wenn über Krebs... Im Fernsehen, in der Zeitung liest man das natürlich schon sehr aufmerksam.“

P06: „Ich bin zum Arzt gegangen, weil ich hier einen Knoten gespürt habe. Und da habe ich schon vorher gewusst, ich habe Krebs.“

P04: „Und da ist plötzlich dieses Bewusstsein gekommen: ‚Du wirst dort nie wieder hinkommen.‘ (...) Und diese körperliche Einschränkung hat mich [unterdrückt Tränen] am ersten Tag, als ich da war, das hat mich schon ganz schlimm erschüttert.“

P05: „Nein, das hörst ja nicht. Das... brauchst zwei, drei Tage... Das kommt dir immer wieder in den Kopf und dann denkst dir: "Ach, das hätte ich noch fragen können, das hätte ich noch fragen können!..." Aber selber... Du bist mit der Krankheit überfordert.“
